# The Science of Homœopathy

**Published:** 1874-07

**Authors:** 


*The Science of Homœopathy; A Critical and Synthetical Exposition of the Doctrines of the Homœopathic School.* By Charles J. Hempel, M. D. New York: Boericke & Tafel, 1874.

We felt highly complimented when the publishers sent us this work, for we felt that they considered us as sitting in the shadow of darkness, and in their kindness had determined to throw upon us the full light of Science as revealed by the law of *Similia similibus curantur.* The author has a tender feeling toward those who practice regular medicine; he offers "a volume which will enable them to acquire the knowledge which they are so much in need of, and which will establish some foundation for their claim of being considered 'Physicians.'"

Surely if Dr. Hempel is an expounder who speaks with authority, the homœopathic school is slowly but surely letting its old dogmas slip away from its grasp. He tells his readers that he does not desire to be exclusive regarding doses, which we suppose to mean that if he cannot control a case of cholera infantum by the thousandth attenuation of mercurius, or the three hundreth attenuation of arsenicum, he considers himself at liberty to pursue some more rational mode.

If Dr. Hempel's production is to be called "Science" commend us to the blissful ignorance of the Hottentot, for we are utterly unable to comprehend what relation his wordy paragraphs about "Cosmic force," "vital princi ple" etc., has to do with the subject under consideration. We shall infer that the author had seen a spirit or had something to do with "spirits" when he wrote the following paragraphs on pages 60 and 61, for it certainly could not have been the unaided production of one human brain.

First, we had this trinity of facts; morbid properties of the tissues or latent capacities for disease developed by the cosmic life-force as individualized in the organism, into concrete diseases characterized be definite symptoms: here we have

Secondly, a similar trinity of facts, namely: the cosmic life-force acting upon germinal principles in the crust of our planet, and developing them into concrete drugs possessed of specific morbific powers.

Believing that there is unity in the system of Creation and that the forces of Creation are summed up in man as a microcosm, I argue that the drug-germs inherent in the crust of the earth, correspond with fully developed diseases. If this proposition be generally true, we have a perfect right to infer from this general truth this most particular application: that a drug which has power to develop in the tissues an approximative image of some particular disease is in curative rapport with it. It seems to me that this inference is irresistible, and that the legitimacy of the Homœopathic law of cures, as a law of Nature, is fully made out by the series of arguments which I have endeavored to present.

It is difficult to part with a theme so sublime, and appealing so powerfully to the mind of every thoughtful worshipper of God, and of the harmonies of His creation. We call Homœopathy a science. So it is; but it is a science of the highest order; it is not only the science of the healing art; it is one of the everlasting and infinite harmonies, of which God's own Providence is the fountain-head and centre.

Homœopathy is a theosophic revelation; it is a philosophic system not fenced in by the limits of a human brain, but which is co-eternal and co-infinite with the Love and Wisdom of the Divine Creator.

When he follows this by the sober declaration on page 77 that "even syphilitic affections are distinctly reproduced by the action of mercury upon healthy tissues." And again by the following (italics our own):

"We desire to state most emphatically that those who deny or overlook the absolute value of drug symptoms; their identity, in numberless cases, with the pathognomonic signs of diseases; the peculiarities which very frequently characterize their action; their occurence or disappearance in *certain positions of the body*, or at *particular periods of the day;* are not inspired by the genius of the homœopathic healing art, and must ever fail in doing justice to its life-saving behests," we close the book in disgust.

